# Update on recent advances in amyotrophic lateral sclerosis

**DOI:** 10.1007/s00415-024-12435-9

**Published:** 2024-05-27

**Authors:** Nilo Riva, Teuta Domi, Laura Pozzi, Christian Lunetta, Paride Schito, Edoardo Gioele Spinelli, Sara Cabras, Enrico Matteoni, Monica Consonni, Eleonora Dalla Bella, Federica Agosta, Massimo Filippi, Andrea Calvo, Angelo Quattrini

**Affiliations:** 1 3Rd Neurology Unit and Motor Neuron Disease Centre, Fondazione IRCCS “Carlo Besta” Neurological Insitute, Milan, Italy; 2 Experimental Neuropathology Unit, Division of Neuroscience, Institute of Experimental Neurology, IRCCS San Raffaele Scientific Institute, Milan, Italy; 3 Department of Neurology, Division of Neuroscience, Institute of Experimental Neurology, IRCCS San Raffaele Scientific Institute, Milan, Italy; 4 Neuroimaging Research Unit, Department of Neurology, Division of Neuroscience, Institute of Experimental Neurology, IRCCS San Raffaele Scientific Institute, Milan, Italy; 5 Vita-Salute Huniversity, Milan, Italy; 6 Istituti Clinici Scientifici Maugeri IRCCS, Neurorehabilitation Unit of Milan Institute, 20138 Milan, Italy; 7 ALS Centre, ‘Rita Levi Montalcini’ Department of Neuroscience, University of Turin; SC Neurologia 1U, AOU città della Salute e della Scienza di Torino, Turin, Italy

**Keywords:** Motor neuron disease, Pathogenesis, ALS genetics, Clinical management, Neuroimaging, Gene therapy, Assistive devices, Gene therapy

## Abstract

In the last few years, our understanding of disease molecular mechanisms underpinning ALS has advanced greatly, allowing the first steps in translating into clinical practice novel research findings, including gene therapy approaches. Similarly, the recent advent of assistive technologies has greatly improved the possibility of a more personalized approach to supportive and symptomatic care, in the context of an increasingly complex multidisciplinary line of actions, which remains the cornerstone of ALS management. Against this rapidly growing background, here we provide an comprehensive update on the most recent studies that have contributed towards our understanding of ALS pathogenesis, the latest results from clinical trials as well as the future directions for improving the clinical management of ALS patients.

## Introduction

Motor neuron disease (MND) covers a heterogeneous group of neurological disorders defined and characterized by degeneration of motor neurons. With an incidence of approximately 2.16 per 100.000 person-years and a median survival time of 3 to 5 years, amyotrophic lateral sclerosis (ALS) is the most common and severe form, involving both lower motor neurons (LMN) and upper motor neurons (UMN), underpinned by a complex interplay between genetic predisposition and environmental factors [1, 2]. Starting from our previous update [3], this review summarizes the recent highlights in ALS research published in the Journal of Neurology and other relevant scientific Journals during the last 60 months.

## Epidemiology

Recent studies confirmed that ALS incidence in European populations is the highest worldwide, probably due to genetic background and high life expectancy in developed regions [2, 4–6]. Moreover, the latest studies reported an increasing incidence of ALS year by year in Ireland, Scotland and some regions of Italy [2, 7–11]. The rising incidence of ALS in these countries may be related to multiple factors, such as the increased ascertainment, a reduced rate of emigration or increasing longevity [2, 9–11]. Conversely, in Asia, the overall incidence of ALS is lower than Europe and North America but, interestingly, the total prevalence is only second to that of West Europe [6], which may be in part linked to genetic differences, such as the lower prevalence of the *C9orf72* hexanucleotide repeat expansion in Asian countries [2, 12, 13]. To increase the knowledge of the ALS genetic puzzle, it will also be crucial to conduct studies on admixed populations or communities with a non-European background [2, 14]. In this respect, preliminary results from the Latin American Epidemiologic study of ALS (LAENALS) corroborated the different genetic predisposition of these populations, supported by a low incidence and prevalence of ALS in these regions [15]. Previous studies initially reported an increased incidence of ALS among males, reporting a male-to-female ratio up to 2:1 [16]. However, more recent studies have refined the estimate of sex ratio and a recent meta-analysis described a male-to-female ratio of approximately 1.3:1, confirming that ALS is slightly more frequent in man. This difference may be due to sex hormones, unequal environmental exposure or sex chromosomes [17, 18]. Several factors have been addressed to explain this variability in sex ratio, such as potential differences in ascertainment strategy, increased life expectancy among females or the type of study (population vs non-population-based studies) [17]. Importantly, a recent study highlights new findings on the relationship between sex and age at onset, revealing a high incidence of males for both youngest and oldest onset and changing ratios at increasing age [17]. This latter observation may be related to the high association between the female gender and the pure UMN phenotype, which is correlated with a long survival [19–21]. Sexual dimorphism indeed entails relevant prognostic implication since man showed an higher weight loss and respiratory function deterioration than women, resulting in a worse prognosis [22]. However, the role of sex is more complex and controversial since it is also recognized that the flail arm phenotype, which is associated with a longer survival, is more common in man [18], and might also implicate differing structural and functional differences in the brain, as highlighted by recent neuroimaging studies [23, 24]. More studies are needed to clarify the interaction between sex, gene and time, which may confer susceptibility to the development of the disease [13, 17, 21].

## Proposed disease mechanisms

In the last few years, further advances have been made in our understanding of MND pathogenesis, a complex process involving a variety of genes and multiple pathways, such as an imbalance of protein homeostasis in neurons, alteration of RNA metabolism, mitochondrial dysfunction and the accumulation of protein aggregates in the cytoplasm of neurons with a toxic gain-of-function [3, 25–28].

Since the first ALS-causing gene Cu–Zn superoxide dismutase 1 (*SOD1*) was discovered almost 30 years ago [29], more than 40 genes have been associated with the inheritance or pathogenesis of ALS. To date, four major genes are known to cover up to 60% of familial forms of ALS and 10–13% of sporadic ALS cases, i.e., *SOD1*, the chromosome 9 open reading frame 72 (*C9orf72*), TAR DNA-binding protein (*TARDBP*) [30, 31], and fused in sarcoma (*FUS*). The *C9orf72* hexanucleotide repeat expansion, discovered in 2011 [32, 33], is the most common genetic cause in European familial ALS (fALS) cases (more than 30%), but also accounts for approx. 7% cases of sporadic ALS (sALS). A great portion of familial FTD cases (approx. 25%) also carried the *C9orf72* pathological expansion, explaining the genetic overlapping of ALS and FTD diseases.15, [34, 35]

Most of *SOD1* disease-causing variants are missense mutations and account for 15–30% of fALS cases and less than 2% of sALS cases [36]. Mutations in *TARDBP* are found in approx. 4% of European fALS cases, whereas *FUS* is more common in Asian fALS cases (6.4% vs 2.8% of European fALS) [37]. Variants in other genes are found in less than 1% of patients [38]. A significant proportion of fALS patients still remain without a genetic diagnosis, suggesting a more complex disease pathogenesis and that other ALS genes may be involved and remain to be discovered [38].

An acceleration in ALS-related gene discovery came in the 2010s with the advent of next generation sequencing (NGS) and more recently, of whole-genome and exome sequencing (WGS/WES, respectively), technologies that facilitated the discovery of numerous genetic variants and enabled large-scale studies to identify ALS risk genes [39]. TANK-binding kinase 1 (*TBK1*) [40, 41] and NIMA-related kinase 1 (*NEK1*) [42] are two examples of genes discovered thanks to these large-scale studies, and subsequently confirmed as ALS-related genes [43, 44]. Broadly speaking, the advent of WGS/WES allows today the detection of more than 250 new genetic association each year, which includes a number of disease relevant variants [45], as well as many variants of uncertain significance (VUS). Interpreting the disease relevance of VUS represents a major challenge as many variants initially reported to be potentially pathogenic, from in silico prediction tools, are later reclassified as benign after additional experimental evidence is accumulated [39]. Loss-of-function (LoF) mutations of the *TBK1* gene are nowadays accepted as the cause of a dominant form of ALS and FTD, potentially explaining up to 4% of fALS cases, including *TBK1* in the ALS-major genes group. These mutations result in an impaired interaction of TBK1 with optineurin and p62, both already implicated in ALS pathogenesis, through a common pathway of autophagy regulation [41, 43, 46–48]. In the last few years, different studies have highlighted a significant enrichment of *NEK1* LoF variants in ALS, and an additional role for the p.Arg261His missense variant in ALS susceptibility. A study conducted in an Italian ALS cohort suggested also a correlation between the presence of *NEK1* variants and the flail arm-ALS phenotype [42, 44, 49].

A loss-of-function mechanism has also been associated with the DnaJ heat shock protein family member C7 (*DNAJC7*), which has been identified as a genetic risk factor for ALS according to a large-scale WES study [50]. Indeed, a functional study has recently shown reduced protein levels in ALS patients carrying a p.R156X variant, suggesting a role of *DNAJC7* in the regulation of protein misfolding, which is a major molecular pathology in ALS, through its interaction with HSP90 and HSP70 [51].

Haploinsufficiency is a disease mechanism shared by different ALS-related genes, such as *TBK1* and *DNAJC7*.[43, 51] A recent study reported a variant leading to small ubiquitin-like modifier (*SUMO4*) haploinsufficiency as a novel potential genetic risk factor for ALS. Recent evidence does point towards a role of the SUMO system in protecting from ALS pathology, by regulating both the assembly and dissolution of the stress granules, possibly acting in a synergistic manner with environmental oxidative stress-related factors [52].

Single-nucleotide variants in the gene encoding Kinesin Family Member 5A (*KIF5A*), a neuronal motor protein involved in anterograde transport along microtubules, have been also recently associated with ALS [53, 54]. Aberrant splicing has been identified as the mechanism by which *KIF5A* mutations cause ALS through a toxic gain-of-function, leading to the aggregation of KIF5A in distal axons and thus to neuronal toxicity. Numerous *KIF5A* mutations found in ALS patients are, indeed, clustered near the splice-site junctions of exon 27 and are predicted to alter the cargo-binding domain of KIF5A [55].

More recently, chloride channel CLIC-like 1 (*CLCC1*) has also been reported as a novel ALS-related gene. The loss of *CLCC1* by a functional mutation was found to cause an ALS-like phenotype in mice [56]. CLCC1 is a transmembrane protein localized on the ER, which maintains ionic homeostasis between the ER and the cytoplasm. Another recent study reported different *CLCC1* variants, all located at the C-terminus of the protein, which are predicted to affect protein stability and present with a similar ALS phenotype, namely an earlier age at onset with rapid progression and cognitive deficits. Thus, *CLCC1* exon 10 might be a potential hotspot for ALS [57]. Interestingly, a study conducted in 2021 proposed the association of variants in serine palmitoyltransferase, long-chain base subunit 1 (*SPTLC1*) with juvenile ALS (namely ALS4), but not with adult-onset ALS, and implicated sphingolipid metabolism as a pathway in motor neuron disease [58]. This study also presented evidence, supported by other studies, that mutations in different genes might influence aspects of the ALS phenotype, as well as the disease progression rate and patient survival. The *C9orf72* repeated expansion, for example, is associated with a faster progression rate while *SOD1* mutations are associated with a slower course [59]. Moreover, the size of the *C9orf72* expansion might represent a modifier of the cognitive phenotype. Indeed, a recent paper highlighted that C9-ALS patients with larger expansions had a more severe cognitive impairment, thereby supporting the emerging hypothesis that *C9orf72* hexanucleotide repeats size might be a modifier of phenotype along the FTLD clinical spectrum [60]. Another recent study suggested an association between the *C9orf72* and an accelerated decline in respiratory function. In addition, this association seemed to be more distinct in spinal-onset male patients [61]. Interestingly, polyglutamine (polyQ) intermediate expansions (24–34 CAG repeats) in the neuronal stress protein Ataxin 1 (ATXN-1) and Ataxin 2 (ATXN-2) genes have been recently reported to be independently associated with an increased risk for ALS [62, 63]. The risk of ALS increases exponentially with allele repeat size until the range known to be associated with spinocerebellar ataxia (SCA1 and 2) risk, even though the age of onset does not seem to be affected [63]. In addition, a study based on an Italian ALS cohort showed that the ATXN-2 intermediate-length polyQ repeat ALS risk correlated with a spinal phenotype and is associated with shorter survival [64].

A trend towards a lower male-to-female ratio in *SOD1* ALS carriers has emerged in a recently published meta-analysis conducted on ALS patients harboring *SOD1* mutations, leading the authors to suggest that differences in sex hormones may have less influence on ALS pathogenesis in the presence of a genetic mutation [13]. Lastly, the presence of variants in more than one ALS-related gene, with a potential contribution of hereditary peripheral neuropathy genes, might also influence the patient's phenotype, supporting an oligogenic model of ALS [65]. Recent reports also hypothesized that *GRN* gene variants could be ALS phenotypic modifiers [66] and that senataxin (*SETX*), the genetic cause of ALS type 4 (ALS4), could be a modifier of *C9orf72* pathology. *SETX* mutations in ALS4, a rare juvenile-onset familial form of ALS, presumably cause a gain-of-function, as loss-of-function mutations in the *SETX* gene are responsible for autosomal recessive Ataxia with Oculomotor Apraxia type 2 (AOA2), and AOA2 carriers do not develop motor neuron disease [67, 68].

## Environmental factors

Various environmental factors have been proposed to be associated with ALS, such as smoking, antioxidants intake, alcohol consumption, body mass index, physical exercise, head trauma, metabolic and inflammatory factors, cancer, and occupational or environmental exposures to electromagnetic fields, metals or pesticides [25, 26, 69–73]. However, to date the only well-established risk factors are represented by age, male gender, and family history [1]. Ref. [74] Previous studies have suggested that certain groups of athletes (especially soccer or American football players) may have an increased risk of ALS. Over the years, several studies tried to associate physical activity with the risk of developing ALS, leading to the hypothesis that the increased risk of ALS may be linked to factors related to sport rather than physical activity alone [75–77]. Indeed, recent studies evidenced that head impact exposure may increase the risk of neurodegenerative disease, in line with the observation that sports like soccer and football are those more linked to the development of ALS [78, 79]. However, other recent studies showed that physical activity in general and other sports such as skiing may be associated with the risk of developing ALS [80–82]. Several putative mechanism have been hypothesized, ranging from oxidative stress to exercise-induced changes in neuron morphology [80]. Nevertheless, most of these studies were retrospective, while prospective or population-based studies found no evidence for an association with the risk of ALS and on the contrary demonstrated that physical activity reduced the risk of ALS mortality [80]. Importantly, these controversies may lead one to hypothesize that head trauma and physical activity may represent a risk factor only in individuals with a specific genetic predisposing background, but to date, there is still a lack of studies investigating the interaction between these factors [83]. Preliminary results in *C9orf72* carriers support this theory, since physical activity may increase the risk of ALS development and may be associated with an earlier age at onset of disease, but more studies are needed to confirm this hypothesis [12, 84, 85]. There are weaker or conflicting data for other putative environmental risk factors [1]. Nevertheless, a recent study on the geographical distribution of presymptomatic ALS patients revealed higher-incidence cluster areas, supporting the idea that exogenous factors may be involved in the ALS pathogenesis [86]. This hypothesis is in line with the results of another recent study focused on ALS incidence among immigrants in Sweden, which found that the risk of ALS was the same as native Swedish only in second-generation individuals, while it was lower in first-generation people [87]. Occupational risk factors have also been considered and a recent study reinforced the notion that veterans may have an increased risk of ALS, especially for Air Force personnel [88]. It remains unclear which factors may increase this risk, given the difficulties in identifying the exact environmental agents, but is supposed that it may be related to specific toxins such as pollutants, heavy metals or infectious agents [89–92].

## ALS phenotype heterogeneity

The ALS clinical spectrum includes extremely heterogeneous and complex phenotypes marked by a varying involvement of upper and lower motor neurons, site of onset and rate of progression [93, 94]. Recognized MND phenotypes include classic, bulbar, flail arm, flail leg, pyramidal and respiratory ALS, primary lateral sclerosis (PLS), characterized predominantly by pure/predominant UMN degeneration and progressive muscular atrophy (PMA), characterized by pure/predominant LMN degeneration [95, 96]. The motor phenotype is so heterogeneous that it may overlap with other diseases of the MND spectrum, such as hereditary spastic paraparesis (HSP) or distal motor neuropathy (dSMA). Noteworthy, recent studies have demonstrated that ALS causative genes are not uniquely associated with a single clinical form but may be responsible for different phenotypes within the MND spectrum, ranging from predominant lower to upper motor neuron involvement. For instance, recent reports have highlighted how such genes as *DCTN1* (dynactin 1) [97–99], *GDAP1* (ganglioside-induced differentiation-associated protein 1) [97, 100, 101], *DYNC1H1* (Dynein, cytoplasmic 1, heavy chain 1) [102, 103], *KIF5A* (Kinesin Heavy Chain Isoform 5A) [102, 104], and *NEFH* (neurofilament heavy chain gene) [105, 106] have been associated with a wide range of phenotypes, ranging from ALS to HSP [107], dSMA or even classic Charcot–Marie–Tooth disease [97, 102, 103, 108–110].

Bulbar ALS (bALS) is more homogeneous both in terms of progression [111] and neuropathological features [112, 113] and is characterized by rapid decline with short survival times (< 2 years post-diagnosis), and a significantly reduced quality of life [114, 115]. Epidemiologically, bALS represents 30% of all ALS presentation [95], and has recently been reported to be more prevalent among German than Chinese patients (35.9 vs. 22.8%) [14]. Pseudobulbar palsy is a variant primarily characterized by impairment of the cortico-bulbar tract that slowly spreads to the limbs: women are reported to be more affected than males, survival is longer and patients are particularly bothered by pseudobulbar affect [116, 117]. Although pseudobulbar palsy may be considered as an ALS variant, it is important to note that a varying degree of pseudobulbar affect (i.e., pathological crying and laughing or emotional lability) may be observed in up to 50% of ALS and PLS patients and has been related to cortico–pontine–cerebellar networks. Pseudobulbar affect may have a considerable impact on quality of life and should be promptly recognized in order to set up appropriate pharmacological interventions [118]. On the other hand, progressive bulbar palsy (PBP) electively involves lower motor neurons of the medulla oblongata nuclei, subsequently spreading to limbs [117]. Although disease progression and spreading is attended, median survival can be longer compared with classic bulbar-onset ALS [119]. Within this context, anti-IgLON5 disease is a proteoform neuro-immunological disorder syndrome, mimicking ALS, characterized by bulbar sign and symptoms, cognitive alteration, gait disturbance and sleep disorders [120]. Interestingly, four IgLON5 IgG seropositive bALS patients with vocal cord paresis at onset have recently been reported [121, 122]. Vocal cord paresis is rarely described as a presenting symptom in bALS, but if present is often found in association with the D90A mutation [123]. Another atypical and rare bALS variant is represented by Facial Onset Sensory Motor Neuropathy (FOSMN), whose hallmark is sensory trigeminal involvement at presentation [124], followed by weakness of the buccal muscles, swallowing disorders and subsequent spread to involve the limbs with cognitive behavioral impairment of a frontotemporal type. The balance of evidence suggests that FOSMN is most likely to be a TDP-43 proteinopathy [125].

Although accounting for a relatively small proportion al ALS patients (3–5%), respiratory onset ALS is a clinical phenotype that needs special mentioning since these patients may be misdiagnosed and referred to neurological attention for a formal diagnosis after the development of respiratory failure or even after being intubated [95, 126]. Notably, a recent study showed that this phenotype is more frequent in older men with predominant lower motor neuron involvement, weight loss, and shorted survival, needing prompt non-invasive ventilation (NIV) adaptation [126, 127].

Notably, a recent study have demonstrated how the cognitive profile in PLS resembles ALS–FTD, without prominent behavioral disturbances [128], in line with a report of a kindred with an association between hereditary PLS and progressive non-fluent aphasia [129]. In addition, an advanced MRI study showed that in PLS the disease burden in the motor cortex is more medial than in ALS, consistent with its lower limb predominance, while the extra-motor profile includes marked insular, inferior frontal, left pars opercularis and cerebellar white and grey matter degeneration, with the postcentral gyrus being relatively well preserved compared with ALS [130–132]. While suggesting a specific profile, these findings still support the notion that PLS lies on the ALS–FTD spectrum. However, the mechanisms underlying disease spread and slow disease progression are likely to be distinct in PLS [20, 128]. Similarly, a recent paper highlighted that in flail arm syndrome, a restricted MND phenotype characterized by progressive, predominantly proximal weakness and atrophy of the upper limbs, clinically covert involvement of the pyramidal tract is similar to classic ALS [18, 133], while another recent paper proposed diagnostic criteria for Mills’ syndrome, a rare MND variant characterized by a slowly progressive, spastic hemiparesis [134].

## Cognitive and behavioral changes in ALS

ALS is a heterogeneous neurodegenerative disorder that can no longer be considered a disease limited to the motor system [25, 26, 135]. While physical deterioration account for the primary symptoms, cognitive and/or behavioral changes are experienced in up to half of patients with ALS and 5–15% of ALS cases meet the criteria for frontotemporal dementia (ALS-FTD) [136–138] with the behavioral variant (bvFTD) mostly represented within ALS-FTD patients [139–141]. However, the neuropsychological deficits in ALS are extremely heterogeneous [135, 138]. Originally construed as a disorder of behavior and executive impairment [142], the neuropsychological profile of ALS is now known also to be associated with alterations in language, social cognition (Table 1) and memory [143], albeit inconsistently [135, 144]. The revised consensus criteria for the diagnosis of cognitive and behavioral dysfunction in ALS has led to re-conceptualization that neuropsychological deficits fall along a spectrum: from no impairment to mild cognitive impairment (ALSci), mild behavioral impairment (ALSbi), both (ALScbi) or ALS-FTD; thus embracing the concept of a frontotemporal spectrum disorder [135]. Specifically, according to guidelines, ALSci is attributed to frontotemporal dysfunction since it is diagnosed when executive and/or language deficits and/or impairment of social cognition occur in non-demented ALS patients, while the ALSbi diagnosis requires the identification of apathy or the presence of at least two non-overlapping, supportive diagnostic features for bvFTD [135, 145]. However, most ALS-FTD patients present with heterogeneous combinations of deficits in behavior, language, or ‘frontal’ cognitive functions [146]. Overall, when present, cognitive deficits and behavioral changes significantly and adversely impact patient survival [147], psychological well-behind [148], consent capacity [149] and caregiver-burden [150]. Interestingly, the presence of ALSci seems to have limited relevance for the patients' and caregivers’ everyday life in comparison to the impact of behavioral alterations, namely apathy and disinhibition [150–153].

**Table 1:** Neuropsychological key concepts

Definition	Neuropsychological assessment and practical implication in ALS
Social cognition (SC) underlies the individuals’ ability to “*make sense of others’ behaviour*” [154]. SC entails a variety of skills, ranging from decoding social signs (e.g., faces and emotional expressions) and drawing inferences on others’ mental or affective states [155]. This natural disposition to interpret others’ mental states and emotions requires the development of a “Theory of Mind” (ToM)	**Assessment:** SC in ALS is typically assessed using paradigms such as theory of mind (ToM) and recognition of facial expressions of basic emotions [156, 157] **Implication for ALS:** Previous research has highlighted challenges among ALS patients in interpreting social cues, such as eye direction, to discern the mental states of others [158]. Recent evidence suggests specific difficulties in recognizing negative emotions such as disgust, anger, fear, and sadness [158, 159], as well as struggles with tasks assessing both affective (emotion, feelings) and cognitive (intention, thoughts) components of ToM [160, 161]. In addition, ALS patients may encounter challenges in processing social versus non-social contexts [162]. Deficits in social cognition are a recognized aspect of the cognitive phenotype of ALS [135, 157]. The particular difficulty in identifying and understanding the feelings and thoughts of others from a self-perspective may be related to behavioral changes such as apathy and diminished awareness, which some patients may exhibit [160]. These findings underscore the need for targeted interventions to address social cognitive deficits in ALS patients, potentially improving their quality of life and interpersonal interactions
Cognitive reserve (CR) refers to differences in ability to cope with aging brain or disease‐related brain changes, facilitating different levels of clinical impairment at similar levels of pathology [163]. Essentially, individuals with higher CR are better able to withstand these changes without experiencing significant cognitive impairment [164]	**Assessment**: indirect measures of CR include premorbid intelligence, educational attainment, occupational complexity, active lifestyle, and bilingualism. They are informative proxies of CR, attempting to represent personal experiences that contribute to the development of CR [165] **Implication for ALS**: recent findings have underscored the complex interplay between CR proxies and cognitive outcomes in ALS [166]. Cross sectional studies showed that higher education correlated with increased pathological burden in medial frontal regions, regardless of cognitive impairment level, suggesting that CR moderates the effect of brain morphology on cognition in ALS [167, 168]. Consistently, lifestyle factors were associated with better-preserved executive functions, verbal fluency, and memory performances [169]. However, longitudinal studies showed that greater educational attainment, occupational complexity, and physical activity were linked to preserved cognitive functioning only at baseline and did not protect cognition over 12 months [170], but only decline in verbal fluency (i.e., an ALS-specific cognitive deficit) [171]. Interestingly, CR could also have a role in motor functional disability in patients with bulbar-onset [169], suggesting that CR can encompass bot cognitive and motor domains. These results motivate future research into CR and practical implications, such as strengthening reserve to delay decline

The extent to which cognitive and behavioral impairment occurs and whether or not it precedes the onset of motor symptoms is still unknown. In ALS-FTD, most patients have FTD features before motor onset, but others developed them at the same time or even after (even if less frequently) motor impairment. In ALS, a presymptomatic or prodromal period of varying duration, during which cognitive and behavioral involvement gradually unfolds, has been postulated in ALSci and/or ALSbi cases [172]. Studies from presymptomatic genetic carriers support this view: for instance, *C9orf72* carriers may manifest dysexecutive and verbal fluency deficits before symptomatic disease onset [173, 174]. Despite two large cohort studies showing a higher frequency of cognitive and behavioral impairment in the later ALS stages [136, 175], it is still debated whether the frequency of neuropsychological changes is different across disease stages. It has been suggested that motor and cognitive components might worsen in parallel [136], and that cognitive worsening is more pronounced in patients with bulbar involvement [175]. But additional factors possibly modifying the clinical expression have been identified, namely *C9orf72* gene mutations [60, 176], cognitive reserve [169] and apolipoproteinE genotypes [177].

From an anatomical perspective, the susceptibility to ALS is not only confined to motor neurons but it also involves extra-motor pathways [26, 178, 179], whose extension and severity seems to be related to the presence and degree of cognitive and behavioral involvement [180, 181]. Recent studies suggest that ALS pathological processes begin in motor neurons and propagate to other regions of the brain in a fashion that is predictable, based on proximity and connection by a corticofugal axonal spread [182–184]. The trajectory of the spreading pattern seems to be consistent with the inclusion and dissemination of phosphorylated 43-kDa transactive response DNA-binding protein (pTDP-43) [183, 185]. This suggests that ALS has a focal onset with subsequent spread along brain connections [186] in the prefrontal neocortex eventually developing “frontal type” cognitive deficits (i.e., executive and social), depending on disease duration and rapidity of propagation [183]. However, the timing of the manifestation of cognitive syndromes does not necessarily follow such sequential staging [187, 188]. A specific pathological course distinct from that of pure-motor (classic) ALS has been found and it is in keeping with the view that cognitive/behavioral involvement might represent a phenotypic marker for distinct ALS subtypes [147, 187].

Another central theme is the recognition of neuropsychiatric symptoms in ALS [189, 190]. Schizophrenia, depression, anxiety, dependence disorders, and psychosis have all been reported in both ALS and ALS-FTD in the years prior to or following diagnosis [191]. A predisposition to neuropsychiatric symptoms is more frequently described in patients with *C9orf72* expansions and presymptomatic *C9orf72* carriers [189]: ALS patients with this expansion exhibit high rates of delusions and hallucinations and may experience psychotic symptoms for years before the onset of motor or cognitive features. It has also been established that ALS patients with a family history of mood disorders or premorbid depressive symptoms have high risk of developing ALSbi, namely apathetic behavior, dysexecutive profile or ALS-FTD [189]. However, it is still unclear if neuropsychiatric symptoms contribute to the spectrum of frontotemporal disorders in ALS as there is considerable variation in the reporting of mood disorders in ALS, ranging from moderate depression to an absence of symptoms [192]. Nevertheless, ALS patients may exhibit symptoms of depression and anxiety (reported in about 30% of patients) at different stages which may reflect a reactions to their condition or alternatively an integral part of the disorders, as a result of frontotemporal and subcortical involvement [191, 193].

Despite the last decades having witnessed a substantial increase in the identification and understanding of frontotemporal dysfunction in ALS, measures designed to evaluate the non-motor features of ALS [194–196] have not been frequently employed in clinical care, research or trial design for ALS [197, 198]. Understanding the diverse clinical presentation of frontotemporal syndromes, their pathological and genetic substrates is crucial for improved early diagnosis, clinical management and the development of therapies tailored for specific ALS sub-groups.

## Extra-motor involvement

Although ALS was initially regarded as selectively affecting the motor system, increasing evidence highlights extra motor involvement, indicating that ALS should be recognized as a multi-system disorder [25, 26, 199]. Ref. [94, 197, 200] In addition to the cognitive involvement, recent studies highlight that ALS may affect other CNS structures, such as the extrapyramidal system. Indeed, a recent prospective study showed that up to 30% of ALS patients may have extrapyramidal features meeting the diagnostic criteria for parkinsonism [201, 202]. These results are in line with another study which found that extrapyramidal deficits with impaired gait initiation in a subset of ALS patients [203]. Moreover, a recent report evidenced that the applause sign, which was initially reported as a specific sign of progressive supranuclear palsy, may also be present in about ~ 10% of ALS/FTLD patients [204]. It is unclear if these manifestations are related to basal ganglia involvement or affection of other brain circuitries and require further investigation [201, 203]. The involvement of cerebellar circuits is another finding that has been consistently—though only recently—highlighted in patients with ALS, especially in those carrying a *C9orf72* gene expansion [205–207], although its role is still debated. A recent study has indicated focal damage to the anterior cerebellar regions in sporadic ALS patients, in contrast with a more posterior damage in *C9orf72* expansion carriers, suggesting that cerebellar pathology might modulate different motor (i.e., dysarthria, coordination and gait deficits) and neurocognitive features (i.e., behavioral dysfunction and deficits in social cognition) according to the genetic background [208]. Intriguingly, focal cerebellar pathology has already been described also in PLS patients, involving especially the middle cerebellar peduncle, where it may be related to the development of pseudobulbar affect [132, 209]. The link between ALS and frontotemporal lobar degeneration (FTLD) is actually well acknowledged, especially in *C9orf72* carriers [60]. A recent study confirmed the early prefrontal involvement in *C9orf72* carriers, firstly suggesting an executive eye movement dysfunction, even in the presymptomatic phase [173]. The increasing interest on retinal involvement in neurodegenerative diseases in recent years also includes ALS with evidence that the retinal nerve fiber layer (RNFL) may be reduced, with an asymmetric involvement and correlating with disease progression [210]. However, these results need to be confirmed, since other reports while confirming the presence of retinal involvement, have not found that they correlate with disease duration or severity [211–213]. More recent studies have focused also on the autonomic nervous system and skin nerve fibers in ALS patients. One such study performed on a small cohort of ALS patients confirmed the high frequency of skin biopsy abnormalities [214]. These results are in line with previous reports, disclosing an association between ALS patients and small-fiber neuropathy, especially among those with spinal-onset or sensory symptoms [200, 215–218]. Moreover, in this study, the authors reported the finding of phosphorylated TAR DNA-binding protein 43 (pTDP-43) deposition in Meissner’s corpuscles in ~ 30% ALS patients, highlighting a potential value of skin biopsy as an ALS biomarker [214]. In addition to small-fiber nerve pathology, recent research found that ALS patients may have autonomic dysfunction. In this work, performed on 102 patients and 41 healthy controls, the authors found an increased rate of autonomic symptoms among ALS patients (~ 70%), especially among those with a bulbar-onset [219]. These results are in line with some reports disclosing a co-occurrence of a rare cardiomyopathy (i.e., Tako-Tsubo syndrome), whose complex pathophysiology may be in part related to sympathetic hyperfunction, especially in bulbar-ALS patients [220]. Further studies are needed to better characterize the extra-motor involvement in ALS patients, which may reflect a specific disease mechanism [221].

## Prognosis

ALS phenotypic heterogeneity is reflected in the variability of ALS prognosis: although approx. one-half of patients die within 30 months of symptom onset, approx. 20% of patients survive between 5 and 10 years [2, 3, 25, 26, 222, 223].

A major determinant of ALS prognosis is disease progression, which is in clinical practice monitored by using the multidomain ALS functional rating score-revised (ALSFRS-R), whose limits have extensively been previously highlighted, but still remains the gold standard for primary efficacy outcomes in clinical trials [25, 224–227]. To overcome these limitations, staging paradigms have been recently proposed such as the King’s [228], and ALS Milano-Torino Staging (ALS-MiToS) [229]. The King’s, based on El Escorial criteria, is more sensitive early in the disease course, while the ALS-MiToS, is based on loss-of-functionality in ALSFRS-R domains and so is better for more advanced disease states [230, 231].

Given the broad distribution of survival, the identification of the key prognostic factors is, therefore, important for appropriate timing of medical interventions and stratification in clinical trials.

Of note, a recent paper pointed out that prognostic factors, which are known to modify the course of disease in Caucasians, apply to Chinese patients as well, despite the apparent differences regarding genotype and clinical phenotype. Specifically, younger age of onset, spinal onset, high BMI and low progression rate were positive prognostic factors in China as well as in Germany [232]. Conversely, long survival has been linked to younger age at onset, increased prevalence of PLS and longer diagnostic delay, absence of the *C9orf72* repeat expansion and increase frequencies of gene variants in *FIG4*, *hnRNPA2B1*, *SETX*, *SQSTM1*, *TAF15*, *VAPB* and the SOD1 p.(Ile114Thr) [233].

Recent data have confirmed the relevance of BMI, metabolism and nutritional status as independent predictors on survival in ALS [234–238]. Within this context, a recent paper has pointed out that body weight changes after diagnosis strongly predicts survival in ALS, and weight gain after diagnosis may improve survival prognosis [239]. In line with this, a hypometabolic or hypermetabolic state have been, respectively, associated with better or worse prognosis [240, 241].

Given that respiratory failure is the main cause of mortality in ALS, measurements of respiratory functionality are integrated not only in clinical practice, but also in ALS prognostic models [12, 242]. However, more recent studies have highlighted that not only slow (SVC) or forced vital capacity (FCV) [243] may be a useful prognostic marker but that other parameters such as arterial blood gas parameters and base excess may independently predict shorter survival in ALS [244, 245]. Within this context, a recent neurophysiological study pointed out that Diaphragmatic CMAP amplitude from phrenic nerve stimulation may also be able to predict functional decline in ALS [246].

In the last few years, in order to overcome the limitations linked to ALS complexity, several prognostic models have been proposed, combining multiple predictors [222, 247–255]. Most models present, however, different methodological limitations, as recently pointed out in a systematic review focused on this topic [247]. More recently, artificial intelligence (AI) and machine learning methods have been adopted in order to develop reliable prediction models, even if their translation into clinical practice is currently still limited [256, 257].

## Diagnostic challenges

Clinical examination remains the cornerstone of ALS diagnosis [258]. The diagnosis of ALS has been traditionally categorized by the El Escorial criteria into various levels of certainty depending on the presence and progressive spread of UMN and LMN signs [259]. Subsequently, the Awaji criteria proposed fasciculations as a clinical and electrophysiologic sign of LMN involvement, aiming at increasing overall diagnostic sensitivity [260, 261]. However, El Escorial and Awaji criteria are hampered by ALS heterogeneity and do not capture the full disease spectrum [261, 262]. In addition, a significant proportion of ALS patients may never attain the criteria for clinically definite ALS [263]. In order to overcome these limitations, the Gold Coast criteria have been recently proposed classifying patients as having or not having ALS, streamlining diagnostic certainty and eliminating confusion from the El Escorial terminology [264]. However, cognitive and behavioral impairment [265] are not included in these criteria [261, 262, 264].

Although an ALS diagnosis is generally fairly simple [261], the false-positive rate has been estimated to be as high as eight to 10% [266], while the false-negative rate approaches 45% [267, 268]. Several disorders, often referred to as ALS mimics, may primarily affect the motor system, simulating ALS. These include Sandhoff disease, adult polyglucosan body disease, Hirayama disease, spinal dural defects associated with CSF leaks and neurogenic calf amyotrophy with CK elevation by entrapment radiculopathy [269–273]. In selected cases, motor nerve biopsy may be useful for an early differential diagnosis of patients presenting with an atypical LMN syndrome [274–276], while motor‑evoked potentials may prove essential in order to detect subclinical UMN involvement in LMN phenotype [277]. Most importantly, differentiation between MND, in particular progressive muscular atrophy (PMA), and multifocal motor neuropathy (MMN) may be extremely challenging. Although MMN has distinctive neurophysiological features and is often associated with anti-GM1 antibodies [278, 279], a recent large case–control study showed that both PMA and MMN, but not ALS and PLS, are associated with an IgM monoclonal gammopathy (8% and 7%, respectively), suggesting that a subset of patients presenting with PMA share pathogenic mechanisms with MMN.[280]

## Biomarkers

The lack of specific biomarkers is recognized as a major limitation in ALS management and clinical research including diagnosis, prognosis and patient stratification in clinical trials. In addition, biomarkers may play a crucial role in assessing the efficacy of potential new treatments and so extensive efforts have been recently made in order to develop potential biomarkers with the aim that they will be specific, repeatable over time and minimally invasive for patients.

In the last years, neurofilament light chain (NfL) and phosphorylated neurofilament heavy chain (pNFH) have exhibited promising diagnostic and prognostic capabilities, making them well suited for integration into clinical workup [281–286]. Moreover, the development of ultrasensitive digital immunoassays (e.g., Simoa™ and ELLA™) able to measure accurately picogram levels have enabled the identification of cutoff values for serum NfL in clinical settings, thus encouraging their routine clinical use as biomarkers [287]. A recent genetic trial involving the antisense oligonucleotide tofersen in *SOD1*-related ALS further confirms the role of NfL as a prognostic biomarker since early reductions of NfL were able to predict the subsequent slowing of ALSFRS-R decline after Tofersen treatment [288]. Other studies have importantly demonstrated that NfL blood levels increase prior to the clinical manifestation of ALS [289–291], suggesting that NfL are valuable biomarkers especially in the earliest phases of the disease and to monitor phenoconversion. All these studies have positioned NfL and pNFH as the most promising biomarkers for ALS, placing them at the forefront of potential integration into clinical practice.

Consistent with the involvement of inflammatory processes in ALS, many studies have explored the role of inflammatory biomarkers in cerebrospinal fluid (CSF) revealing alterations in the concentrations of cytokines, chemokines, and complement proteins (CIT). In particular, recent studies have revealed that chitotriosidase (CHIT1), chitinase-3-like protein 1 (YKL-40, or CHI3L1), and chitinase-3-like protein 2 (YKL-39, or CHI3L2) demonstrate a significant increase in their concentrations in the CSF of ALS patients compared with mimicking conditions [292, 293]. The levels of urinary neopterin, a marker of cell-mediated inflammation, have been found to be higher in ALS patients compared with both patients with other neurodegenerative diseases and ALS-mimics, supporting a role for neopterin as a diagnostic biomarker [294, 295]. Urinary neopterin concentration reflects the severity of ALS, underscoring neopterin’s potential as a prognostic biomarker in ALS.207.

Given the recent advances in our understandings of the impact of metabolism in ALS disease progression [296, 297], recent studies have explored the potential role of metabolic biomarkers. A recent case–control study suggested that high triglycerides may be associated with longer survival, whereas higher levels of cholesterol were associated with a higher mortality [298], while another study explored the role of irisin, a peptide hormone released by muscle and involved in metabolism, showing higher levels of irisin in ALS patients with impaired metabolic status compared to normo-metabolic ALS patients and healthy subjects [299].

Recently, circular RNAs (circRNAs) are emerging as a novel candidate biomarkers in ALS patients. In a recent study exploring a microarray expression profile of circRNAs in leukocyte, samples from ALS patients have suggested that specific circRNAs candidates have value as potential novel blood-based biomarkers for ALS, displaying AUC values exceeding 0.95 in ROC curve analysis [300]. Further studies regarding circRNAs and their association with ALS should be done in order to validate these results.

Despite numerous studies aimed at validating different potential markers in ALS disease, none is currently adopted in the clinical settings. In a future perspective, a panel of different biomarkers reflecting various aspects of the disease, would be more suitable for routine clinical practice and would allow one to glean more information on the different mechanisms involved in ALS.

## Advances in neuroimaging

Over the last decade, neuroimaging techniques have seen dramatic improvements, opening up exciting opportunities to investigate new aspects of CNS structure and function in ALS patients [117, 301, 302]. In fact, neuroimaging provides an ideal tool to explore quantitative, reproducible measures of UMN impairment and is sensitive to extra-motor brain involvement that frequently accompanies the development of the cognitive and behavioral impairments seen in ALS [303]. These techniques have proven their utility for phenotypic characterization, disease monitoring and patient stratification in relation to clinical outcomes, as well as providing new insights into pathogenesis.

Conventional brain MRI protocols provide only limited accuracy for supporting an MND diagnosis by the demonstration of T2-weighted hyperintensity within the corticospinal tract (CST) [304]. A large consecutive series of ALS patients reported that susceptibility-weighted imaging showed the presence of a hypointense signal in the cortical band along the precentral gyrus (i.e., the “motor band sign”) in approximately 80% of ALS patients [305], although the specificity of this radiological sign is still to be determined. Advanced computational neuroimaging approaches have been used to describe in greater detail patterns of grey (GM) and white matter (WM) structural damage characterizing specific groups of ALS patients. For example, CST metrics have been used to discriminate aggressive forms of ALS from more slowly progressing cases [306–308]. Patients with faster clinical progression showed more severe cortical thinning of the left precentral gyrus and fractional anisotropy reduction of the CST relative to those with slower rate of progression [307]. Recently, cluster analysis based on both GM and WM measures was used to identify, through a data-driven approach, two ALS subtypes, characterized by different clinical profiles and degree of frontotemporal cortical involvement, with clear prognostic implications [205]. Another recent study evaluating morphometric modifications and diffusion tensor (DT) MRI alterations showed distinctive patterns of GM and WM involvement in ALS and PLS, with greater damage to extra-motor cortical and cerebellar structures in PLS patients [130]. In MND patients with predominant LMN involvement, DT MRI metrics were also able to distinguish fast- from slow progressors, based on the greater involvement of the CST and extra-motor WM tracts in patients with a more rapid disease progression [309–311]. On the other hand, another study has suggested that the flail arm syndrome, a phenotypical variant of ALS, may show a similar degree of CST involvement as to that seem in patients with a ‘classical’ ALS presentation [133]. Subtle structural brain alterations have been shown to occur even in the earliest phases of the disease, as presymptomatic carriers of a *C9orf72* expansion demonstrated significant, selective thalamic and focal hippocampal volume reductions [207, 312].

Structural neuroimaging has also shown its utility to track ALS progression. A study investigating cortical thickness changes over time in different ALS phenotypes found a significant decline of cortical thickness in frontal, temporal, and parietal regions over time [313]. Effects were independent of the clinical subtype, with the exception of the precentral gyrus: the LMN ALS variants demonstrated the highest rates of cortical thinning in the precentral gyrus, the UMN-dominant subjects exhibited intermediate rates of atrophy, and the classical ALS patients exhibited no real change as the atrophy of the precentral gyrus seen in classical ALS is at floor on the first assessment, resulting in a lack of further atrophy over time [313]. A probabilistic fiber tractography study estimated structural connectivity changes after three months in patients with ALS [314]. While CST damage did not worsen over time, DT MRI changes were observed in the occipito-temporal pathways, again suggesting an early involvement of motor networks with subsequent extra-motor damage [314]. Recently, a tract-based staging scheme has been proposed to assess cerebral progression of ALS pathology in vivo, supported by the fact that the progression of WM alterations across tracts using DT MRI was associated with clinical disease severity in patients with ALS [315].

In addition to brain alterations, spinal MRI has also been shown to have role in the prognostic stratification of ALS patients. A recent multicenter study demonstrated that multiple stepwise linear regression models based on clinical variables and a combination of cervical spinal cord cross-sectional areas, diameters and DT MRI parameters can provide an accurate prediction of motor capacity as early as at 3 months from diagnosis [316]. Another recent study exploited the combined acquisition of both brain and spinal cord MRI sets to test the competing hypotheses of anterograde (i.e., dying-forward) vs retrograde (i.e., dying-back) neurodegeneration in ALS [317]. The analysis of volumetric and diffusion data in a common domain identified step-changes in tissue damage measures between the cranial descending corticospinal tract and C1 spinal cord level, and between C5 and C6 cord levels, with an apparent cranio-caudal gradient [317], although further validation of these data is needed to draw definitive conclusions about this issue.

In addition to MRI, positron emission tomography (PET) imaging has also drawn interest as a tool to visualize disease-related metabolic and molecular changes occurring in the brain of individuals with ALS with different clinical phenotypes. For example, ^18^F-FDG PET has demonstrated a pattern of increasingly severe frontal hypometabolism associated with the degree of cognitive and behavioral impairment in a large cohort of patients with ALS, with additional cerebellar damage in those with comorbid FTD [318], whereas a metabolic signature of relative damage to the lateral areas of the precentral gyri was found in patients with a pure bulbar involvement, relative to those with a pure spinal involvement [319]. The severity of frontotemporal hypometabolic patterns also showed a prognostic significance, being associated with greater functional impairment and shorter survival in patients with ALS and PLS [320]. Moreover, genetic background was found to influence ^18^F-FDG PET findings in ALS, as hypometabolism in frontotemporal regions, basal ganglia, and thalami could be detected in patients carrying a *C9orf72* gene expansion since the presymptomatic stages [321]. By utilizing radiotracers targeting various molecular pathways implicated in ALS pathology, PET imaging also enables the non-invasive assessment of disease-related alterations in vivo. Among the most intensively studied molecular targets in the last years, the translocator protein (TSPO) of the outer mitochondrial membrane has been mostly considered a marker of activated microglia. Increased local uptake of TSPO tracers (such as ^11^C-PK11195 and ^11^C-PBR28) was consistently detected in the motor cortex, prefrontal cortex, pons, and thalami of sALS patients, colocalizing with structural abnormalities of both grey and white matter and showing significant correlations with clinical measures of UMN burden [322, 323].Similar findings were shown in ALS patients with pathogenic genetic variations in the *SOD1* gene, even in the presymptomatic phases [324].

All these findings suggest that an advanced multiparametric neuroimaging approach has the potential to improve our understanding of the neural substrates underlying the clinical impairment seen during the ALS disease course.

## Advances in pharmacological treatments

Despite considerable efforts and numerous well-designed clinical trials, Riluzole remains the only drug with any evidence for a demonstrated ability to increase survival in ALS patients, even if the mechanisms of action are not yet fully understood [325–327]. Recent studies also confirmed that Riluzole is well tolerated with a suggestion that a prolonged duration of riluzole intake correlates with longer median survival [328, 329].

Intravenous edaravone, a free radical scavenger, is another approved drug in Japan, U.S., Canada, South Korea and Switzerland, as it showed a positive effect on disability progression in a subgroup of patients [330]. However, a prospective evaluation conducted by the Italian EDARAVALS study group showed that in the Italian cohort, Edaravone, although overall well tolerated, had no significant effect in slowing disease progression or respiratory function decline [331]. These data are in line with a longitudinal MRI study showing no significant changes in fractional anisotropy (FA) values of corticospinal tracts and cerebral cortex thickness (CT) in ALS patients treated with Edaravone [332].

Different disease mechanisms have been targeted during these years, often adopting the strategy of repurposing compounds already used in clinical practice for the management of other conditions, based on data from ALS preclinical models [333–335]. Acetyl‑l‑carnitine has recently been restudied in a retrospective cohort after a previous phase 2 trial showing efficacy on self-sufficiency, ALSFRS-R total score and forced vital capacity (FVC) [336]. Compared with the non-treated patients, the group receiving the 1.5 mg dose had a higher number of subjects still alive at 24 months after baseline, while no differences were detected with the 3 mg dose group. A confirmatory phase 2/3 trial is planned in 2024 (NCT06126315). Tauro-urso-deoxycholic acid (TUDCA), a small molecule with preclinical evidence of anti-apoptotic effects, was shown in two independent phase 2 trials, alone and in combination with sodium phenylbutyrate (PB), to be able to modify disease course in ALS patients, as further confirmed by a retrospective analysis of real-life patients [337, 338]. Based on the results of these phase 2 trials, the FDA has already approved Relyvrio® (PB and taurursodiol), event if preliminary announcement of topline results has reported that both the *Phoenix trial and the* TUDCA-ALS study did not meet the prespecified primary endpoints, even if the data from these two independent phase 3 trials on the effects of TUDCA and PB in ALS are sill pending [339, 340].

A phase two trial evaluated the effect of Perampanel (an anti-epileptic drug) in reducing disability and increasing survival. However, this study showed no effect of the treatment on the different endpoints, with a high number of adverse events and a more rapid decline of ALSFRS-R in the treatment group [341].

Several trials have targeted neuroinflammation. A pilot trial on 7 patients treated with leukapheresis followed by intravenously Treg infusions and IL-2 injections or placebo provided insights on a possible efficacy of this well tolerated treatment, especially on ALS patients with a neuroinflammatory profile [342]. Another therapeutic attempt in this field has been recently done with a small cohort of ALS patients who underwent autologous hematopoietic stem cell transplantation, but further studies should be carefully evaluated based on the low benefit/risk balance [343]. Rapamycin has been investigated in a multicenter, randomized, double-blind trial, in 63 ALS patients at the dose of 2 mg/m^2^/day, 1 mg/m^2^/day and placebo (EUDRACT 2016–002399-28; NCT03359538). Despite the primary outcome not been achieved, rapamycin was able to decrease mRNA relative expression and plasmatic protein levels of the pro-inflammatory cytokine IL-18, increasing the percentage of classical monocytes and memory switched B cells [344].

Two phase 3 trials testing drugs that increase muscle contraction, Levosimendan [345] and Reldesemtiv [346], failed to demonstrate efficacy on disease outcomes, despite a good safety profile.

One of the most promising therapeutic innovations in the fields of neuromuscular disorders and ALS is represented by the use of antisense oligonucleotides (ASOs) [334]: a phase 3 trial of Tofersen, an ASO targeting *SOD1* mutations, showed important effects on disease biomarkers (SOD1 and neurofilaments), despite mild clinical efficacy [288]. The open-label extension is still ongoing, even if the FDA has already approved its use. Other phase I/III studies are evaluating the efficacy of the ASO Jacifusen (also known as ION363), directed at patients with mutations in the FUS (fused in sarcoma) gene, which is responsible for certain forms of very rapid, early-onset disease [347]. Also within the target therapy represented by ASO technology, it is worth mentioning the currently ongoing phase I study aimed at evaluating the efficacy of ASO BIIB105 in silencing the Ataxin 2 (*ATXN-2*) gene, a known disease modulator associated with an accelerated phenotype, in patients with mutations in both *ATXN-2* and sALS [348]. Despite these encouraging results, an attempt to exploit the ASO technology (Tadnersen) in patients with the pathogenic repeat expansions in the *C9orf72* gene did not show the hoped-for efficacy in a preliminary phase I trial [349].

Among the non-pharmacological interventions that have been tried, a trial of high calorie therapy showed efficacy in increasing weight and BMI, though other disease outcomes were not reached [350], while another study assessed the impact of physical therapy on ALS, comparing aerobic, flexibility and muscle-strengthening exercises vs muscle-strengthening exercises alone [351].

## Clinical management

In the absence of a cure, the cornerstones of the management of patients with ALS focuses on symptoms control [352]. These treatments may not only alleviate symptoms but also maintain quality of life and improve survival, with greater benefit for patients managed in specialized ALS clinics by a multidisciplinary team, able to provide integrated palliative care strategies [242, 353–357]. Symptomatic care in ALS includes a wide range of patient-tailored interventions addressing pain, emotional lability, anxiety and depression, sleep disturbances, constipation and including physiotherapy and adaptive aids. Prevalence of muscle cramps has been estimated to be as high as 44–55% in ALS patients. As recently reviewed, therapeutic options include quinine sulfate, tetrahydrocannabinol, vitamin B-complex, diltiazem, natridrofuryl oxalate, mexiletine, carbamazepine and leveteracitam as well as non-prescription approaches such as muscle stretching [358, 359]. Interestingly, a small case series recently demonstrated that radiotherapy treatment of sialorrhea, which affects up to 25% of patients [360], may be feasible, efficient and safe, even in patients requiring non-invasive ventilation (NIV) [361].

Dysphagia and respiratory failure, which is the main cause of mortality for ALS patient, represent two crucial areas of symptomatic interventions for ALS patients [352]. Malnutrition is a negative prognostic factor [362] and aspiration pneumonia one of most feared causes of morbidity and mortality in ALS, and so current practice guidelines recommend gastrostomy feeding for patients with severe dysphagia [242, 363]. The two main methods of gastrostomy insertion are percutaneous endoscopic gastrostomy (PEG) and radiologically inserted gastrostomy (RIG), even when per-oral image-guided gastrostomy may be possible. Even though it has been suggested that RIG may be safer than PEG, in particular in patients with respiratory failure [364, 365], there is to date little evidence to indicate which is better and what is the optimum timing for the procedure. Indeed, a recent prospective observational study showed that PEG was a safe procedure even in patients with low FVC [366], an observation that was confirmed by another study proposing a modified approach in order to carefully select high-risk patients [367]. These results were further confirmed by another large prospective cohort study showing that the three methods seemed to be equally safe in relation to survival and procedural complications [368]. However, both studies agree that PEG might be less beneficial when delayed. Further studies, and preferably randomized controlled trials are needed in order to gain better evidence for the nutritional management and use of gastrostomy in ALS patients.

Respiratory failure is not only the main mortality cause in ALS, but may also be the presenting feature, in this instance requiring careful differential diagnosis to exclude other neuromuscular and non-neuromuscular disorders is needed.[369] Recommended pulmonary tests are spirometry (including Supine FVC), polysomnography, arterial gas analysis and sniff nasal inspiratory pressure (SNIP), which has recently been shown to be a potential prognostic factor of tracheostomy or death during the early phase of disease [370]. While a recent open-label, randomized controlled trial has shown that addition of diaphragm pacing to standard care was associated with decreased survival in patients with ALS [371], NIV has been shown to improve quality of life and survival in patients with ALS [372], even if there is still no consensus on the exact timing of NIV initiation or cough augmentation techniques [373, 374]. In patients with substantial bulbar impairment, the efficacy of NIV may be more limited; however, a recent study has demonstrated objective sleep and nocturnal respiratory outcomes in both non-bulbar and bulbar patients [375]. Moreover, NIV has also been reported to significantly increase survival by 19 months in patients with ALS-bulbar onset [376]. In more advanced disease states, invasive ventilation via tracheostomy is an option for prolongment of survival [377]. In the more advanced stages, palliative strategies, including respite care, have been demonstrated not only to provide a positive effect not only to meet the needs of people with ALS but also for their care partners [378].

## Emerging assistive technologies for the management of amyotrophic lateral sclerosis

People with ALS face numerous challenges due to the progressive nature of the disease, leading to a rapid physical decline, which restricts mobility and impairs all activities of daily living. This includes speaking, eating, dressing, walking, and writing [379]. The relentless decline in these functions can lead to feelings of sadness, hopelessness, worthlessness, anxiety, and guilt. Despite the physical limitations, patients are aware of their limitations. This disease of losses raises a deep concern among caregivers and patients themselves in preserving autonomy, self-control, and decision-making for as long as possible. Emerging technologies for the management of ALS patients range from multidisciplinary teleconsults (for monitoring the dysphagia, respiratory function, and nutritional status), to robotics. The COVID-19 pandemic created a need to accelerate the development and implementation of such technologies in clinical practice, to improve the daily lives of both ALS patients and caregivers [380–382].

**Assistive technologies (ATs)** can support patients in preserving autonomy and control along with slowing disease progression. They are of great value to ALS patients, since their use may help to overcome severe functional limitations [383]. Autonomy and self-determination play a crucial role in the lives of ALS patients and can partly be maintained by the implementation of ATs and devices. There are many technology options available to support persons with neurodegenerative conditions, either mainstream or specifically designed products [384].

One of the key areas where ATs provide support is communication, also known as **Augmentative and Alternative Communication (AAC)**. As speech intelligibility declines, support in communication becomes important in ALS management. Everyday digital technologies of the last decade, such as smartphones, tablet devices, and the Internet, may be used to assist persons with neurodegenerative conditions allowing them to perform daily tasks; such approaches include using voice-activated commands to control the environment and text-to-speech to communicate verbally [385]. AAC ATs also include brain–computer interfaces and eye tracking systems. For people with ALS, having access to an ecosystem that integrates multi-professional assistance and ATs, particularly AAC resources, has been shown to be essential in preserving communication and interaction skills and enhancing quality of life and survival as the disease advances [386]. ATs and devices can support ALS patients in their mobility, such as through powered wheelchairs and helping them control their domestic environment, and thus foster social participation and autonomy [383, 384].

**Telehealth ATs** may have a significant role in managing ALS. Potential applications include:

1. Telehealth technologies for remote monitoring of dysphagia, respiratory function, and nutritional status, which can increase the effectiveness of ALS management [380, 383, 387, 388].

2. Tele-rehabilitation for delivering physical therapy and other rehabilitative services to patients in their own homes. Therapy sessions can be conducted virtually via video calls, allowing patients to receive therapy from the comfort of their own homes [389].

3. Digital personal assistants that can be used to assist persons with neurodegenerative conditions in performing daily tasks, such as using voice-activated commands to control the environment [383, 384].

**3D printing**, also known as additive manufacturing, is a technology that also has the potential to significantly improve the management of ALS, addressing the challenges associated with the disease. 3D printing can be used to create customized assistive devices tailored to the specific needs of each ALS patient, ranging from modified utensils for eating to custom-fit braces and mobility aids. It can be used to create prosthetics and orthotics that are lightweight, comfortable, and tailored to fit the patient perfectly. This can help improve mobility and independence for ALS patients. Finally, 3D printing can also be used to create specialized medical equipment that can help in the management of ALS, such as modified respiratory equipment or specialized seating systems that improve comfort and mobility [390]. Despite its potential, there are several challenges associated with the use of 3D printing in ALS management: 1. limited material selection; 2. cost of pre- and post-processing; 3. cost of system equipment; 4. acceptance of technology among patients and carers that depends on various factors such as perceived skills and competencies in using the device, expectations, trust, and reliability; 5. regulation and standardization as 3D-printed medical devices must meet the same safety and efficacy standards as traditionally manufactured devices [391]. However, these challenges are not insurmountable. With continued research and development, as well as collaboration between healthcare providers, engineers, and patients, 3D printing has the potential to play a pivotal role in transforming the management of ALS.

**Wearable devices**, such as smart-tracker or smartwatch, and smartphone can track ALS disease progression and may serve as novel clinical trial outcome measures [392–394]. Several wearable devices collecting data daily on physical activity measures have demonstrated statistically significant changes over time with correlations to the ALS functional rating scale-revised (ALSFRS-RSE) and the Rasch Overall ALS Disability Scale (ROADS) [395]. Interestingly, an international survey on patient perspectives on digital healthcare technology in care and clinical trials for ALS showed that most patients had a positive attitude toward the use of digital technology in both care and clinical trial settings [396].

**Assistive robotics** can help ALS patients to perform daily tasks, thereby enhancing their autonomy and quality of life [380]. For instance, a soft robotic wearable developed by researchers from the Harvard John A. Paulson School of Engineering and Applied Sciences (SEAS) and Massachusetts General Hospital (MGH) was able to provide significant upper arm and shoulder support and improved mobility for those with ALS [397]. Some assistive robots are equipped with communication aids and can be customized to fit the specific needs of each ALS patient, which can make them more effective and comfortable to use [379].

**Neural interfaces** are being explored for their potential to restore lost functions in ALS patients. Brain–computer interfaces (BCIs) use neural signals for computer control and may allow people with late-stage ALS to communicate even when conventional technology falls short. BCIs are designed to restore voluntary motor control to paralyzed people by converting intent-to-move nerve impulses from the motor cortex into a digital signal. These technologies commonly use machine learning to interpret the nerve impulses, collected from tiny electrodes implanted in the brain, and transform them into specific digital actions [398].

In recent years, we have seen great progress in the development and validation of implanted BCIs, which place neural signal recording electrodes in or on the cortex [379]. The Wyss Center for Bio and Neuroengineering is planning to launch a trial of its wireless brain–computer interface (BCI), called ABILITY, in people to enable them to communicate using only their thoughts [399]. In a case study, a NeuroKey-based BCI is being tested in a man with a fast-progressing form of ALS, who advanced to a locked-in state and could no longer use assistive devices to communicate [400].

ATs can, therefore, help ALS patients overcome severe functional limitations, allowing patients to retain autonomy and control as their disease progresses^1^.[379] However, despite these benefits, there are challenges and limitations associated with the use of ATs.

1. Physical and environmental barriers: ALS patients may face physical and environmental barriers that limit the effectiveness of ATs.

2. Financial and resource constraints: the cost of ATs can be prohibitive for some patients, limiting their access to these technologies.

3. Poor policy implementation: in some cases, policies related to the provision and use of ATs may not be effectively implemented.

4. Societal ignorance: lack of awareness and understanding about ATs among society at large can also pose challenges.

5. Technical challenges: acceptance of technology among patients and carers depends on various factors such as perceived skills and competencies in using the device, expectations, trust, and reliability.

While ATs have a significant positive impact on ALS management, it is important to address these challenges to make these technologies more accessible and effective for all patients. Moreover, there is a pressing need for further research in this field to address the remaining challenges and so allow us to continue advancing these promising technologies.

## Ethical issues in motor neuron disease

Being a relentlessly fatal disease with no curative therapy currently available, there are many ethical issues in ALS care, from the diagnosis, disease course to the latest stages of disease, as recently reviewed [401]. Despite the fact that many healthcare providers and physicians may find it difficult and stressful [402, 403], ALS patients generally welcome the opportunity to discuss end-of-life issues with their physician [404, 405]. PEG and NIV decision-making processes are complex and individual, comprising patient-centric factors and external factors, including the roles played by healthcare professionals, family information provided and concepts of timing of interventions [406]. Although there is no consensus on timing, it is suggested that options for respiratory support and end-of-life issues be discussed when the patient displays symptoms of hypoventilation [407]. However, end-of-life discussions, including gastrostomy, NIV and tracheostomy invasive mechanical ventilation (TIV) are often delayed for a number but not necessarily justifiable reasons, potentially leading to poor patient management and unplanned crisis interventions [408]. Recent studies demonstrated that caregivers and the general public significantly underestimate the QoL of ALS patients and overestimate the patients’ rate of depression, while the desire to shorten life was significantly lower in ALS patients compared to what healthy subjects thought the patient would wish. Moreover, of those with undecided or negative attitudes, 10% changed their attitude towards life-sustaining treatments during the following year, emphasizing the need for unbiased patient perspectives in order to secure patient-centered decision-making [409, 410], even if the potential executive and behavioral impairments linked to ALS raises further inevitable questions [411].

Many socio-cultural, ethical, legal and financial issues may influence the use of TIV in ALS patients, as reflected by the varied percentage of use between countries, ranging from 0% of patients from the UK, 1–14% in the USA, 11% in northern Italy, to 27–45% in Japan [412, 413]. The ethical implications of TIV have recently been analyzed accordingly to the four core principles of healthcare ethics: beneficence, nonmaleficence, respect for patient autonomy, and justice [414]. However, there are many specific and challenging aspects to be considered within the context of ALS. The choice of TIV is indeed a critical milestones as it may imply survival in a locked-in state [414, 415]. Hence, TIV in ALS affects not only the patient but also the next of kin and the professional caregivers, rendering highly relevant to discuss potential positive and negative consequences also for these other stakeholders [414, 415]. Respect for the patient’s right to accept or refuse medical interventions is the mainstays of modern healthcare. Prerequisites are competence and being informed [149]. However, cognitive and behavioral impairments seen in ALS make it questionable whether these requirements are in place in all circumstances, which is highly relevant to patient autonomy and decision-making [265, 414, 416]. Indeed, the recent awareness of potential executive and behavioral impairment in ALS patients represents a further ethical question, emphasizing the need to develop new methods of neuropsychological assessment suitable to the more advanced stages of disease [149, 409, 417].

## Conclusions

ALS remains a relentlessly progressive and fatal disease and the only approved disease-modifying drug has a modest effect in slowing disease progression. Symptomatic and palliative care, provided in a multidisciplinary context, still remains the cornerstone of ALS management [326]. However, significant progress has been accomplished in the identification of new genetic factors and pathomechanisms underlying this disease, giving new hope for the development of gene therapy and novel mechanistic therapeutic approaches.
